# ARHGEF37 overexpression promotes extravasation and metastasis of hepatocellular carcinoma via directly activating Cdc42

**DOI:** 10.1186/s13046-022-02441-y

**Published:** 2022-07-22

**Authors:** Xin Zhang, Liangliang Ren, Junhua Wu, Rongni Feng, Yunyang Chen, Ronggang Li, Meimei Wu, Mingzhu Zheng, Xing Gui Wu, Wanjun Luo, Hongle He, Yanming Huang, Miaoling Tang, Jun Li

**Affiliations:** 1 Clinical Experimental Center, Jiangmen Key Laboratory of Clinical Biobanks and Translational Research, Jiangmen Central Hospital, Jiangmen, 529030 China; 2 Department of Hepatobiliary, Pancreatic and Splenic Surgery, Jiangmen Central Hospital, Jiangmen, 529030 China; 3 Department of Biochemistry, Zhongshan School of Medicine, Sun Yat-sen University, Guangzhou, 510080 China; 4 Department of Pathology, Jiangmen Central Hospital, Jiangmen, 529030 China; 5 Cancer Center, The First Affiliated Hospital, Sun Yat-sen University, Guangzhou, 510080 Guangdong China

**Keywords:** Extravasation, Rho guanine nucleotide exchange factor, HCC, Metastasis

## Abstract

**Background:**

The extravasation capability of hepatocellular carcinoma (HCC) cells plays a vital role in distant metastasis. However, the underlying mechanism of extravasation in HCC lung metastasis remains largely unclear.

**Methods:**

The expression of ARHGEF37 in human HCC specimens and HCC cell lines was examined by quantitative RT-PCR, western blot, and immunohistochemistry (IHC) analyses. The biological roles and mechanisms of ARHGEF37/Cdc42 in promoting lung metastasis were investigated in vitro and in vivo using cell lines, patient samples, xenograft models.

**Results:**

In the current study, we found that Rho guanine nucleotide exchange factor 37 (ARHGEF37) was upregulated in human HCC samples and was associated with tumor invasiveness, pulmonary metastasis and poor prognosis. Overexpressing ARHGEF37 significantly enhanced the extravasation and metastatic capability of HCC cells via facilitating tumor cell adhesion to endothelial cells and trans-endothelial migration. Mechanistically, ARHGEF37 directly interacted with and activated Cdc42 to promote the invadopodia formation in HCC cells, which consequently disrupted the interaction between endothelial cells and pericytes. Importantly, treatment with ZCL278, a specific inhibitor of Cdc42, dramatically inhibited the attachment of ARHGEF37-overexpressing HCC cells to endothelial cells, and the adherence and extravasation in the lung alveoli, resulting in suppression of lung metastasis in mice.

**Conclusion:**

Our findings provide a new insight into the underlying mechanisms on the ARHGEF37 overexpression-mediated extravasation and pulmonary metastasis of HCC cells, and provided a potential therapeutic target for the prevention and treatment of HCC pulmonary metastasis.

**Supplementary Information:**

The online version contains supplementary material available at 10.1186/s13046-022-02441-y.

## Background

Hepatocellular carcinoma (HCC) is the third leading cause of cancer-related death globally [1]. Despite continuous progress and development in clinical detection and treatment strategies to improve prognosis in terms of overall survival, the occurrence of metastasis has increased prominently [2, 3]. HCC is prone to intrahepatic and extrahepatic metastases, with extrahepatic metastasis occurring in 13.5–42% of patients with HCC [4, 5]. Specifically, the prognosis of HCC patients with extrahepatic metastasis is poor, with a one-year survival rate of less than 25%, and a median survival time of only 4.9–7 months [4, 6]. Lung metastasis is the most common sites of extrahepatic disease, accounting for 34–58%, and being one of the major problems in HCC patients because of limited therapeutic strategies [5, 7, 8]. Thus, understanding the molecular mechanism of pulmonary metastasis of HCC is of great importance to prevent the early initiation of metastasis in patients with HCC and to develop therapeutic strategies in patients with advanced HCC.

Hematogenous dissemination to the pulmonary capillary network is the presumed mechanism of HCC cell spread in pulmonary metastasis [5]. While tumor cells use various strategies to survive the hostile intravascular environment, their metastatic potential eventually depends on the process of extravasation, which involves adhesion to endothelial cells, modulation of the endothelial barrier permeability, and trans-endothelial migration [9, 10]. It has been demonstrated that Rho GTPases plays vital roles in the extravasation of cancer cells [11]. Depleting several Rho GTPases significantly reduced the adhesion of prostate cancer cells to the endothelium and subsequent metastasis towards the lungs in vivo [12]. Additionally, it has been proposed that melanoma cell B16F10 extravasation and lung metastasis is increased by activating integrin α5β1-mediated Rac1/p21 activated PAK1 signaling, which further increased the permeability of the endothelial barrier [13]. Moreover, during extravasation, the Rho GTPases RhoA and cell division cycle 42 (Cdc42) become activated in invadopodia, which provide a motile force to guide the tumor cells through the endothelium of the capillary walls [14]. However, the mechanism underlying extravasation in the pulmonary metastasis of HCC is unknown.

Rho family guanine nucleotide exchange factors (Rho-GEFs/ARHGEFs) are a family of proteins implicated in the activation of small GTPases by cycling from the inactive GDP-bound state to the active GTP-bound state [15]. Several members of the subfamily, including ARHGEF5 [16], ARHGEF15 [17], and ARHGEF9 [18] were shown to activate a number of GTPase, including RhoA and Cdc42. Accumulating evidence has demonstrated that multiple mammalian GEFs specific for the Rho GTPases contribute to cancer cell migration and invasion by enhancing the loading of GTP onto Rho proteins [19, 20]. In the present study, we found that upregulated expression of ARHGEF37 was correlated positively with high risk pulmonary metastatic of HCC. Furthermore, we demonstrated that overexpression of ARHGEF37 increased the Cdc42-GTP level in HCC cells, and enhanced the extravasation and lung metastatic capability of HCC cells through promoting the formation of invadopodia, consequently resulting in disruption the interaction between endothelial cells and pericytes. Therefore, our results provide a new insight into the underlying mechanisms on the ARHGEF37 overexpression-mediated extravasation and pulmonary metastasis of HCC.

## Materials and methods

### Cell lines

Hepatocellular carcinoma cell lines Hep3B2.1-7 (Hep3B), Huh7, and MHCC97H, human embryonic kidney cells (HEK) 293 T were grown in Dulbecco’s modified Eagle’s medium (DMEM) (Gibco, Grand Island, NY, USA) supplemented with 10% fetal bovine serum (Gibco, Grand Island, NY, USA), and were tested for mycoplasma contamination.

The human lung microvascular endothelial cells (HMVEC-L) were purchased from Cell Application, Inc. (Cat. #540-05a, Cell Application, Inc., San Diego, CA) and cultured with the microvascular endothelial cell growth medium (Cat. #111-500, Cell Application, Inc., San Diego, CA) according to the manufacturer’s instructions.

The human lung pericytes were isolated from an adjacent normal tissue in lung cancer patient as previously described [21, 22]. Briefly, tissue was minced and digested in HBSS buffer containing collagenase I (300 U/ml) and dispase (5 U/ml). The suspension was filtered (70 μm), centrifuged (350×g, 10 min), and washed with DMEM + 10% FBS. The dissociated cells were plated on 0.2% gelatin-coated dishes in human Pericyte Medium (ScienCell Research Laboratories, Carlsbad, CA). After expansion, cells were negatively selected by CD45, CD31, and CD326 magnetic beads (Miltenyi Biotec Inc. San Diego, CA) to deplete leukocytes, endothelial cells, and epithelial cells, respectively. Pericytes were collected for labeling with PE-conjugated anti-PDGFR-β (clone REA363; Miltenyi) and anti-PE magnetic microbeads (Miltenyi biotec, Germany) and passed through a magnetized column. Retained pericytes (PDGFR-β^+^) were cultured in human pericyte medium (ScienCell Research Laboratories, Carlsbad, CA).

The enhanced green fluorescent protein (EGFP^+^)-HCC cells were generated by lentiviral infection using GFP expressing lentiviral plasmid and selected for 10 days using 400 μg/mL neomycin. Furthermore, the EGFP^+^-HCC cells stably expressing *ARHGEF37* cDNA or *ARHGEF37* shRNA were established by lentiviral infection using pSin-EF2-*ARHGEF37* or pSuper-retro-*ARHGEF37* shRNA and selected for 10 days with 0.5 mg/mL puromycin.

### Tissue specimens and immunohistochemistry (IHC)

All clinical HCC specimens, including 250 paraffin-embedded surgical non-metastatic HCC tissues and 70 intrahepatic-metastatic and 45 extrahepatic-metastatic biopsy specimens, and 10 freshly collected HCC samples, including 5 surgical HCC tissues and 5 metastatic biopsy specimens, used in the current basic research study were histopathologically and clinically diagnosed. This basic research study complied with all relevant ethical regulations involving human participants. The clinical information regarding the samples is summarized in Table S1–S4. Prior patient consent and approval were obtained from the Institutional Research Ethics Committee of the Jiangmen Central Hospital and the First affiliated hospital of Sun Yat-sen University (Approval number: 2022-51).

IHC staining for protein expression of ARHGEF37 in HCC samples further quantitatively analyzed using the mean optical density (MOD), defined as the mean of immunostaining intensities per positive pixels in a specimens according previously described [23–28]. Briefly, the stained sections were evaluated at × 400 magnification by using the SAMBA 4000 computerized image analysis system assisted with Immuno 4.0 quantitative program (Image Products International, Chantilly, VA), and 10 representative staining fields of each section were analyzed to verify the MOD, which represents the strength of staining signals as measured per positive pixels. A negative control with each batch of staining was used for background subtraction in the quantitative analysis. The MOD data were statistically analyzed by using the t test to compare the average MOD difference between different groups of tissues, and *P* < 0.05 was considered significant.

### Plasmids, lentiviral infection, and transfection

The human *ARHGEF37* cDNA and the *CDC42* cDNA were PCR-amplified from *ARHGEF37* plasmid, purchased from GeneCopoeia (EX-Y4343-M35, GeneCopoeia, China), and CDC42 plasmid, purchased from ViGene Biosciences (OENM-001791, WZ Biosciences Inc., China), and subcloned into pSin-EF2 vector. The serially truncated fragments of the human *ARHGEF37* were amplified by PCR and cloned into the pcDNA3.1 vector, which including F1 (residues 1-250), F2 (residues 250-675), F3 (residues 1-500), F4 (residues 120-675). Short hairpin RNAs (shRNAs) targeting *ARHGEF37* were cloned into the pSuper-retro vector. All primers and oligonucleotides used in plasmid construction are listed in Table S5 and S6. Transfection of small interfering RNAs (siRNAs) or plasmids was performed using the Lipofectamine 3000 reagent (Thermo Fisher Scientific, Waltham, MA, USA) according to the manufacturer’s instructions.

### In vivo metastasis assays

Male BALB/c-nu mice (5–6 weeks of age, 18–20 g) were purchased and housed in specific pathogen-free facilities on a 12 h light/dark cycle. All experimental procedures were approved by the Institutional Animal Care and Use Committee of Sun Yat-Sen University and the approval number was SYSU-IACUC-2022-000322. For tail intravenous injection, the indicated luciferase-expressing cells (5 × 10^5^) were resuspended in 0.1 mL phosphate-buffered saline (PBS) and inoculated into the lateral tail vein of nude mice. To determine the effect of ZCL278 treatment on tumor metastasis, mice were intraperitoneally injected with dimethyl sulfoxide or ZCL278 (20 mg/kg of body weight) every other day for 28 days. We used an In Vivo Imaging System (Xenogen, Alameda, CA, USA) to monitor the metastasis of these transduced cells in mice at the indicated time points. At the end of observation, the mice were killed by cervical dislocation and their lungs were collected to count surface metastases. Extracted lungs were embedded in paraffin and subjected to hematoxylin and eosin staining was performed on sections from paraffin-embedded samples for histological evidence of the tumor phenotype.

### Extravasation analysis in vivo

The in vivo extravasation assays were based on previously described methodology [29–31]. Briefly, at 4 or 24 h after intravenous injection of the indicated EGFP^+^-HCC cells, the mice were anesthetized with sodium pentobarbital (50 mg/kg), and then longitudinally cut the skin and subcutaneous tissues from the abdomen to the chest region with surgical scissors. The pleural cavity was opened with surgical scissors and forceps to fully expose the heart and lungs, and the superior vena cava (SVC) and inferior vena cava (IVC) were tied with sterile surgical sutures to prevent the backflow of perfusion solution during lung perfusion. Furthermore, the left ventricle wall was cut with surgical scissors to open a fissure (about 2-4 mm) to drain the perfusion solution from the lungs. Then the right ventricle was washed with 1x PBS to remove the drained solution with constant suction during lung perfusion until the lungs turn from a reddish color to completely pale, and lungs were perfused and fixed with 2% paraformaldehyde. Lung sections were acquired and immediately imaged using fluorescence microscopy to monitor the location and relative amounts of these transduced cells in mice at the indicated time points.

### RNA extraction and real-time PCR

Total RNA was extracted from the indicated cells using the Trizol reagent (Life Technologies, Carlsbad, CA, USA) according to the manufacturer’s instructions. Real-time PCR was performed using the Eastep® qPCR Master Mix Kit (Promega, Madison, USA) under the following condition, which involved a pre-heating step at 95 °C for 2 min, 40 cycles at 95 °C for 15 s, 60 °C for 30 s. The qPCR primers were designed with the assistance of the Primer Express v 2.0 software (Applied BioSystems, Foster City, CA, USA). Expression data were normalized to the geometric mean of housekeeping gene *GAPDH* (encoding glyceraldehyde-3-phosphate dehydrogenase) to control the variability in expression levels and calculated as 2^- [(Ct of gene) – (Ct of GAPDH)]^, where Ct represents the cycle threshold for each transcript [32]. All primers are listed in Table S6.

### Chemical reagents

ZCL278 was purchased from Selleck Chemicals (Houston, TX, USA). Human recombinant Cdc42 protein was purchased from Abcam (Cambridge, MA, USA).

### Immunoblotting (IB) analysis

Immunoblotting analysis was performed according to a standard protocol with the following antibodies: anti-ARHGEF37 (HPA053487) and anti-Flag (F3165) (Sigma-Aldrich, St. Louis, MO, USA); anti-phosphorylated (p)-PAK1) (phospho S204) (ab79503) and anti-PAK1 (ab131522) antibodies (Abcam, Cambridge, MA, USA); anti-Cdc42 (CST#2466S), anti-N-cadherin (CST#13116S), and anti-Connexin-43 (Cx43) (CST#3512S) antibodies (Cell Signaling technology, Danvers, MA, USA); anti Cdc42-GTP (26905), anti Ras-GTP (26903), and anti RhoA-GTP (26904) antibodies (NewEast Biosciences; King of Prussia, PA, USA), and anti-GAPDH (60004-1-Ig) antibodies (Proteintech, Rosemont, IL, USA).

### Co-immunoprecipitation (Co-IP) assay

Cells grown in 100-mm culture dishes were lysed using 500 μL of lysis buffer [25 mmol/L HEPES (pH 7.4), 150 mmol/L NaCl, 1% NP-40, 1 mmol/L EDTA, 2% glycerol, and 1 mmol/L phenylmethylsulfonyl fluoride]. After being maintained on ice for 30 minutes, the lysates were clarified by microcentrifugation at 12,000 rpm for 10 minutes. To preclear the supernatants, the lysates were incubated with 20 μL of agarose beads (Calbiochem, San Diego, CA, USA) for 1 h with rotation at 4 °C. After centrifugation at 2000 rpm for 1 min, the supernatants were incubated with 20 μL of antibody-cross-linked protein G-agarose beads overnight at 4 °C. The agarose beads were then washed six times using wash buffer [25 mmol/L HEPES (pH 7.4), 150 mmol/L NaCl, 0.5% NP-40, 1 mmol/L EDTA, 2% glycerol, 1 mmol/L phenylmethylsulfonyl fluoride]. After removing all the liquid, the pelleted beads were resuspended in 30 μL of 1 M glycine (pH 3), after which, 10 μL 4 × sample buffer was added, the samples were denatured, and then separated using sodium dodecyl sulfate polyacrylamide gel electrophoresis for immunoblotting analysis.

### Far-Western analysis

Far-Immunoblotting was performed by using the proteins immunoprecipitated by anti-Flag antibody and human recombinant Cdc42 protein. Briefly, the proteins were separated by SDS-PAGE, and were transferred onto a PVDF membrane. Membranes were then preincubated in 10% skimmed milk for 1 hour at 4 °C. As indicated, recombinant Cdc42 protein was added at 3 μg/mL and incubated at 4 °C for 18 hours. After extensive washing six times with TBST, the membrane was subjected to immunoblotting analysis using indicated antibodies.

### Pericyte–epithelial cell (EC) co-culture model

HMVEC-L cells and pericytes were incubated for 30 min at 37 °C with 8 μM Cell-Tracker Green CMFDA and Cell-Tracker Red CMPTX dyes (Thermo Fisher Scientific, Waltham, MA), separately. The cells were then washed using 1× PBS, and incubated with fresh complete cell culture medium for an additional 24 h. In the 2D co-culture model, HMVEC-Ls and pericytes were seeded in 8-well Lab-Tek chamber slides at densities of 20,000 and 6600 cells/well (ratio HMVEC-Ls-pericytes 3:1), respectively. Cell monolayers were processed for N-cadherin (CST#13116S, Cell Signaling technology, Danvers, MA) or Cx43 (CST#3512S, Cell Signaling technology, Danvers, MA) immunofluorescence staining 24 h later. The secondary antibodies were donkey anti-rabbit IgG (H + L) conjugated with Alexa Fluor 647 (A-31573, Thermo Fisher Scientific, Waltham, MA). Cell nuclei were counterstained and mounted with antifade mountant with 4′,6-diamidino-2-phenylindole (DAPI) (Thermo Fisher Scientific, Waltham, MA). The images were captured using the AxioVision Rel.4.6 computerized image analysis system (Carl Zeiss, Jena, Germany).

### Monolayer adhesion assay

The monolayer adhesion assay was performed in a parallel plate flow chamber kit (Cat#31-001, GlycoTech, Gaithersburg, MD, USA) as previously described [33, 34]. The kit contains 4 parts, including: (1) base plate with an entrance and exit port through which cells and media are perfused, (2) a glass or plastic slide plate on which the substrate or cellular monolayer is placed, (3) a gasket that controls the chamber diameter, and (4) a vacuum outlet so that the apparatus can be held in place. HMVEC-L cells were cultured on 24-well and allowed to grow to full confluence. Then, the monolayers were stained with 8 μM Cell-Tracker Red CMPTX dyes (Thermo Fisher Scientific, Waltham, MA) and inserted into a flow chamber. The indicated HCC cells were resuspended (1 × 10^5^ cells per mL) in serum-free culture medium with 0.88 mM fibrinogen and 2.5 mM CaCl_2_ and loaded into a syringe pump. The dynamic adhesion assays were performed at a shear stress in the range of 0.1–4.0 dynes cm^− 2^ according to the manufacturer’s manual. The tumor cells adhering to the monolayers were imaged under a fluorescence microscope and the average number of adherent tumor cells in five random fields at × 200 magnification was calculated.

### Trans-endothelium migration assay

HMVEC-L cells were seeded in 24-well Transwell inserts with a pore size of 8 μm and grown for 2 days until full confluence. The indicated ARHGEF37-dysregulated or control EGFP^+^-HCC cells (5 × 10^5^) were added into the apical chamber. The basolateral chambers were filled with 600 μL culture medium with 10% fetal bovine serum, which was used to suspend the tumor cells in the apical chambers. After incubation for 24 h, the apical side of the apical chamber was scraped gently with cotton wool. Only the migrated EGFP^+^-HCC cells were detected by fluorescence microscopy and were counted from 10 random fields of × 200 magnification.

### Invadopodia gelatin degradation assay

To assess the capability of HCC cells to form invadopodia and degrade the matrix, a QCM Gelatin Invadopodia Assay (ECM670, Merck Millipore, Darmstadt, Germany) was performed according to the manufacturers’ protocol. Briefly, slides were pre-coated with 0.2% w/v poly-L-lysine (20 min at room temperature), and then fixed with 0.5% glutaraldehyde (15 min at room temperature). Following incubation with a gelatin mixture (Fluorescein-labeled and unlabeled gelatin 1:5, 10 min at room temperature), the slides were sterilized with 70% ethanol. Then, cells were added onto labelled-gelatin-coated sides and cultured in the dark under standard tissue culture conditions for 24 h. After fixation with 3.7% formaldehyde, the cells were stained with TRITC-phalloidin and DAPI for 1 h, respectively. In invadopodia experiment, the number of HCC cells forming invadopodia was quantified in 15 microscope fields (63×) randomly and percentage of invadopodia forming cells was assessed. In ECM degradation experiment, 15 microscope fields (40×) imaged randomly and the percentage of degraded area was quantized using ImageJ software (National Institutes of Health) and normalized to the number of nuclei in that area, which was represented as “% degradation per cell”.

### Cdc42-GTP pull-down assay

Cdc42 activation was examined using a Cdc42 Activation Assay Kit (ab211163, Abcam, Cambridge, MA) that uses GST-PAK1-PBD fusion protein agarose beads to selectively isolate and pull-down the active form of Cdc42 from endogenous lysates following the manufacturer’s instructions. GST-PAK1-PBD fusion protein is the p21 binding domain (PBD) of PAK1 that expressed as a GST-fusion protein and coupled to agarose beads. GST-PAK1-PBD fusion protein is used to bind the activated form of GTP-bound Cdc42, which can then be immunoprecipitated with glutathione resin. Briefly, cells were harvested in cell lysis buffer, and the indicated cell lysate was loaded with GDP or GTPγS and incubated with PAK1 p21-binding domain-Agarose beads as negative or positive controls, respectively. One mg of protein lysate in a total volume of 1 mL at 4 °C was immediately precipitated using 40 μL of PAK1 p21-binding domain beads for 60 minutes with rotation. After washing, the beads were resuspended and processed for immunoblotting.

### Statistical analysis

Statistical tests for data analysis included log-rank test, Chi-square test One or Two-way analysis of variance (ANOVA) and Student’s two-tailed t test. Survival curves were plotted by the Kaplan–Meier method and compared by the log-rank test. The metastasis free survival was measured from the data of random assignment to the date of first evidence of recorded clinical metastasis-intrahepatic metastasis and/or extrahepatic metastasis confirmed by imaging or histologic evidence-was censored at the date of last follow-up. The cut-off values for high- and low- expression of ARHGEF37 proteins of interest were chosen based on a measurement of heterogeneity using the log-rank test with respect to metastasis free survival. The significance of various variables for survival was analyzed by univariate and multivariate Cox regression analyses. Statistical analyses were performed using the SPSS 18.0 statistical software package (SPSSInc., Chicago, Ill., USA) and GraphPad Prism 8 (GraphPad Inc., La Jolla, CA, USA). Data represent mean ± SD. *P*-values of 0.05 or less were considered statistically significant.

## Results

### Overexpressed ARHGEF37 correlates with pulmonary metastasis of HCC

**Fig. 1:**
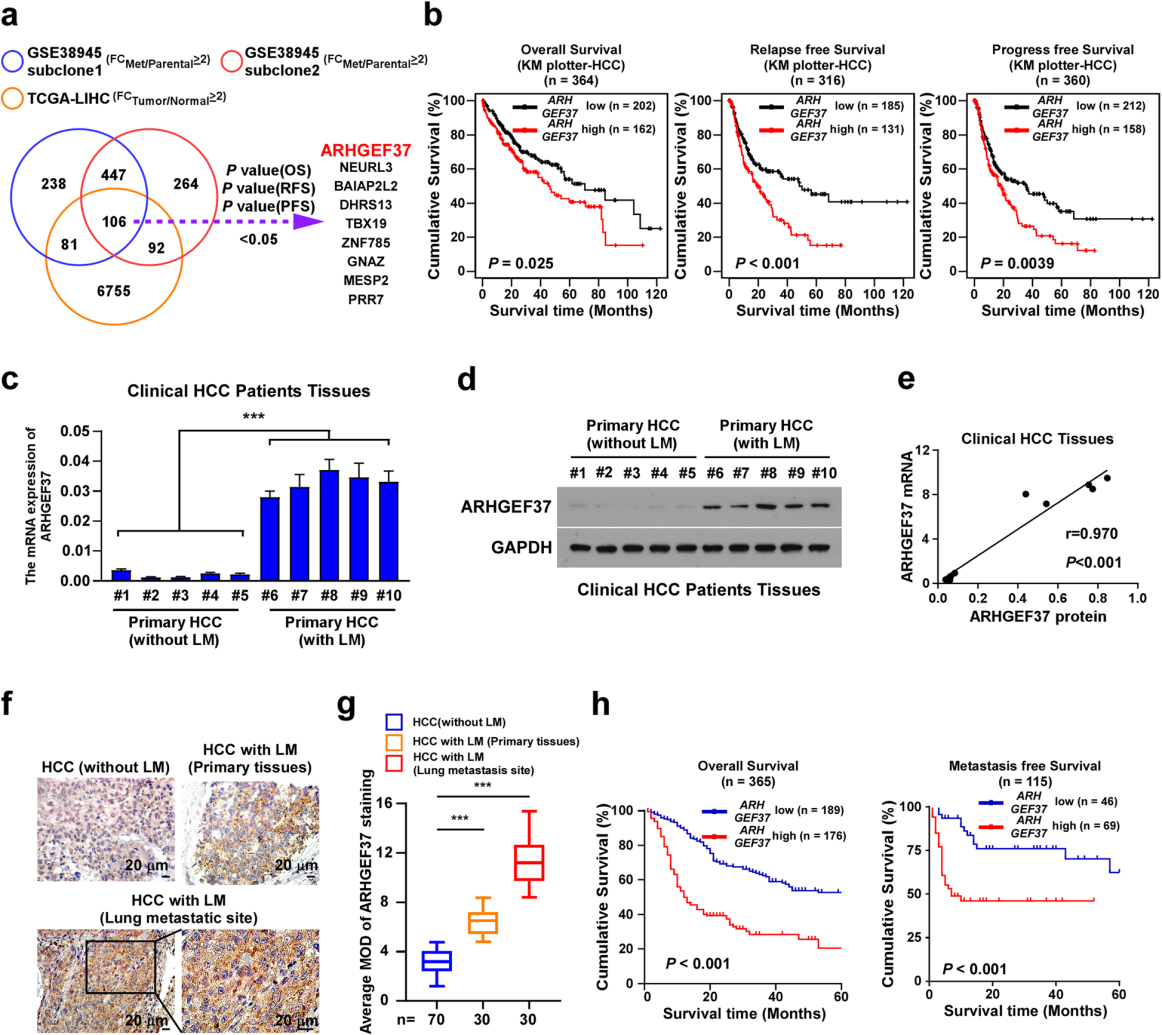
Overexpressed ARHGEF37 correlates with pulmonary metastatic of HCC. **a** The Venn diagram displays the genes that differentially expressed (fold-change > 2, *P* < 0.05) among the public datasets GSE38945 and The Cancer Genome Atlas (TCGA) dataset. **b** Statistical analysis of overall (*n* = 364, *P* = 0.025; left), relapse-free (*n* = 316, *P* < 0.001; middle) and progression free (*n* = 360, *P* = 0.0039; right) survival in patients with HCC with low or high *ARHGEF37* expression in the KM-plotter dataset. **c** qRT-PCR analysis of the mRNA level of *ARHGEF37* in clinical HCC tissues without lung metastasis (LM) (*n* = 5) and primary HCC tissues with lung metastasis (*n* = 5). *GAPDH* served as a loading control. **d** Immunoblotting analysis of ARHGEF37 levels in clinical HCC tissues without lung metastasis (*n* = 5) and primary HCC tissues with lung metastasis (*n* = 5). GAPDH served as the loading control. **e** Correlation analysis of the mRNA and protein expression of ARHGEF37 in clinical HCC tissues. **f-g**) Representative images (**f**) and quantification (**g**) of ARHGEF37 expression in HCC tissues without lung metastasis (*n* = 70), primary HCC tissues with pulmonary metastasis (*n* = 30), and matched HCC lung metastasis tissues (*n* = 30). Scale bar: 20 μm. **h** Kaplan–Meier survival analysis of the association of ARHGEF37 protein levels with overall (*n* = 365, *P* < 0.001; left) and metastasis-free (*n* = 115, *P* < 0.001; right) survival in patients with HCC. Data of panel **d** is derived from three independent experiments, and each error bar in panel **c** represents the mean ± SD of three independent experiments. *** *P* < 0.001

HCC is highly invasive and is prone to extrahepatic metastasis, which occurs preferentially in the lung [35]. To decipher the molecular mechanism contributing to the high invasiveness of HCC, we analyzed two public datasets (GSE38945-Subclone1 and GSE38945-Subclone2) of pulmonary metastasis in HCC and intersected with TCGA HCC dataset. We identified 554 genes that were differentially expressed between the pulmonary metastasis foci samples and the primary tumor samples among those datasets (Fig. 1a; fold-change > 2; *P* < 0.05). Interestingly, online public Kaplan-Meier Plotter database revealed that the expression levels of only nine genes were significantly elevated in HCC tissues and were related negatively to the overall, relapse-free, and progress-free survival of patients with HCC (Fig. S1a and Fig. 1b). Among them, the biological role of *ARHGEF37* in pulmonary metastasis of HCC remains largely unknown. Therefore, we focused on ARHGEF37 for further investigation.

To validate the correlation of ARHGEF37 expression with HCC lung metastasis, we first examined the expression levels of ARHGEF37 in small cohort of freshly collected primary HCC tissues that experienced pulmonary metastasis (*n* = 5) and non-metastatic primary HCC tissues (*n* = 5). As show in Fig. 1c and d, the mRNA and protein levels of ARHGEF37 were significantly higher in the primary HCC that metastasized than in non-metastatic HCC tissues. Corresponding, the expression levels of the ARHGEF37 mRNA and protein were positively correlated in clinical tissues from patients with HCC (Fig. 1e, r = 0.970, *P* < 0.001). Furthermore, IHC assay revealed that the ARHGEF37 protein level was markedly increased in primary HCC tissues that experienced pulmonary metastasis (*n* = 30) and further elevated in HCC tissue at metastatic site (n = 30), compared with that in non-metastatic HCC tissues (*n* = 70) (Fig. 1f and g). Moreover, statistical analysis indicated that patients with high ARHGEF37-expressing HCC had significantly shorter overall survival and metastasis-free survival than those with low ARHGEF37-expressing HCC (Fig. 1h, *P* < 0.001, *P* < 0.001; and Table S1-S4). Taken together, these results suggest that ARHGEF37 overexpression is associated with the poor clinical outcome of HCC.

### Overexpressing ARHGEF37 enhances pulmonary metastatic capability of HCC cells in vivo

**Fig. 2:**
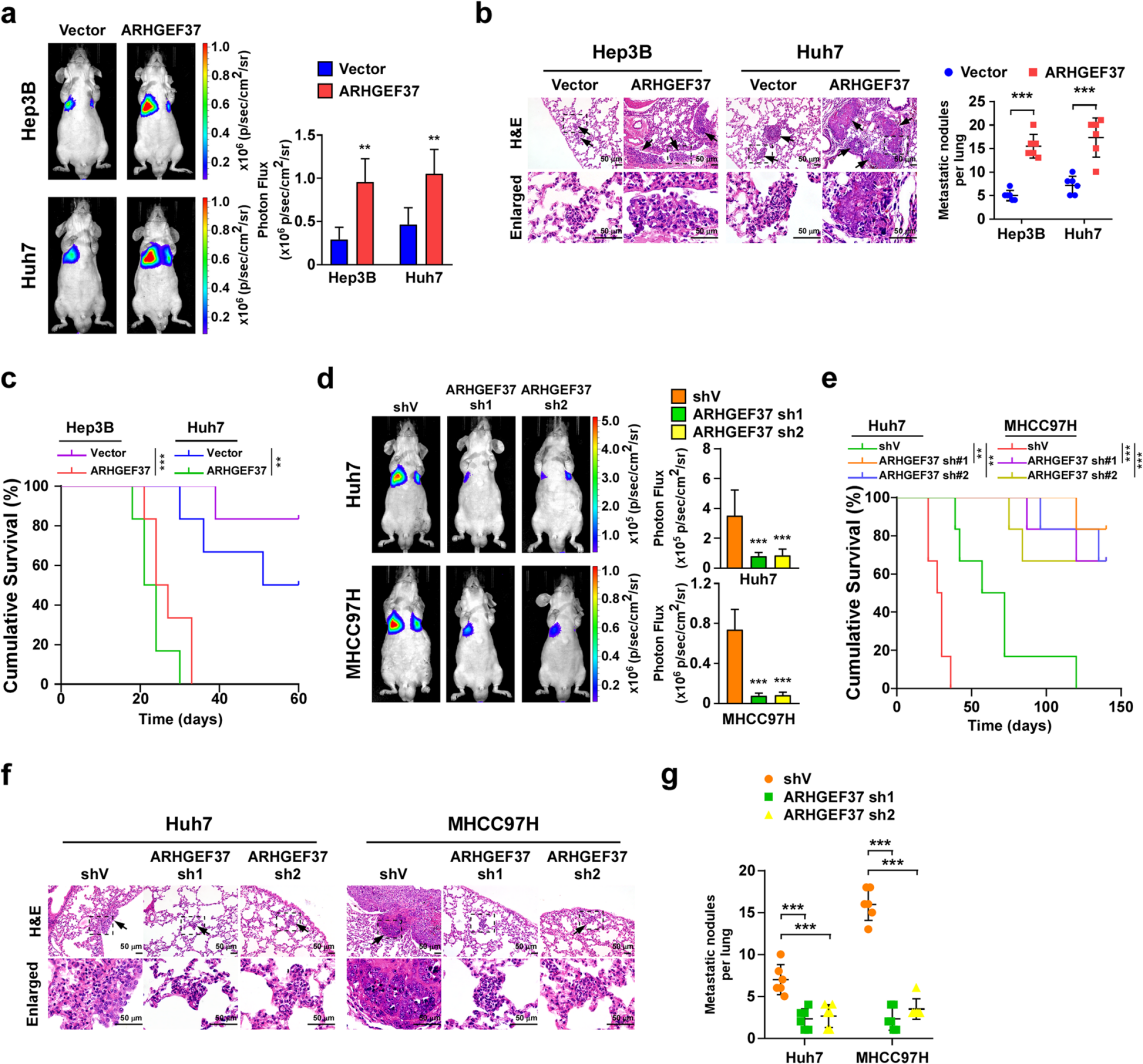
Overexpressing ARHGEF37 enhances pulmonary metastatic capability of HCC cells in vivo. **a** Left: Representative bioluminescence images of pulmonary metastasis in mice after tail vein intravenous injection with vector and HCC cells stably overexpressing ARHGEF37. The color scale bar depicts the photon flux emitted from the mice and pulmonary metastasis (*n* = 6). Right: The statistical results of the luminescence signals for lung metastasis. **b** Representative images and quantitation of the metastatic nodules in the lungs. Scale bars: 50 μm. **c** Kaplan–Meier survival curve of mice from the indicated experimental groups (*n* = 6/group). **d** Left: Representative bioluminescence images of pulmonary metastasis in mice after tail vein intravenous injection with shV- and stable ARHGEF37-silenced HCC cells. The color scale bar depicts the photon flux emitted from the mice and pulmonary metastasis (*n* = 6). Right: The statistical results of the luminescence signals for lung metastasis. **e** Kaplan–Meier survival curve of mice from the indicated experimental groups (*n* = 6/group). **f-g** Representative images and quantitation of the metastatic nodules in the lungs. Scale bars: 50 μm. Each value in panel **a**, **b**, **d**, **g** represents the mean ± SD of six mice/group. ** *P* < 0.01; *** *P* < 0.001; ns represents not significant

To further explore the role of ARHGEF37 in HCC pulmonary metastasis, both Hep3B and Huh7 HCC cancer cell lines stably overexpressing ARHGEF37, or Huh7 and MHCC97H HCC cancer cell lines stably expressing ARHGEF37 shRNAs were established depending on the basic expression of ARHGEF37, respectively (Fig. S2a-S2c). Then the control and ARHGEF37-transduced or ARHGEF37-silenced HCC cells were inoculated directly into the tail veins of nude mice for lung metastasis assays. As shown in Fig. 2a, b and Fig. S2d, markedly larger signals of lung metastasis developed in the ARHGEF37-transduced group, accompanied with increased number of lung metastatic nodules, compared with that in the vector group. Importantly, overexpression of ARHGEF37 in the xenograft HCC cells significantly reduced the overall survival of the mice (Fig. 2c). Consistently, compared with the control mice, the ARHGEF37-silenced HCC cell-injected mice exhibited lower signals of lung metastases and experienced prolonged overall survival (Fig. 2d, e and Fig. S2e). Hematoxylin and eosin staining revealed that the metastatic foci in the Huh7- and MHCC97H- shARHGEF37 group were dramatically decreased in lung tissue sections (Fig. 2f and g). Therefore, these results indicate that ARHGEF37 plays vital role in promotion of pulmonary metastasis in HCC in vivo.

### ARHGEF37 facilitates endothelial adherence and trans-endothelial migration of HCC cells

**Fig. 3:**
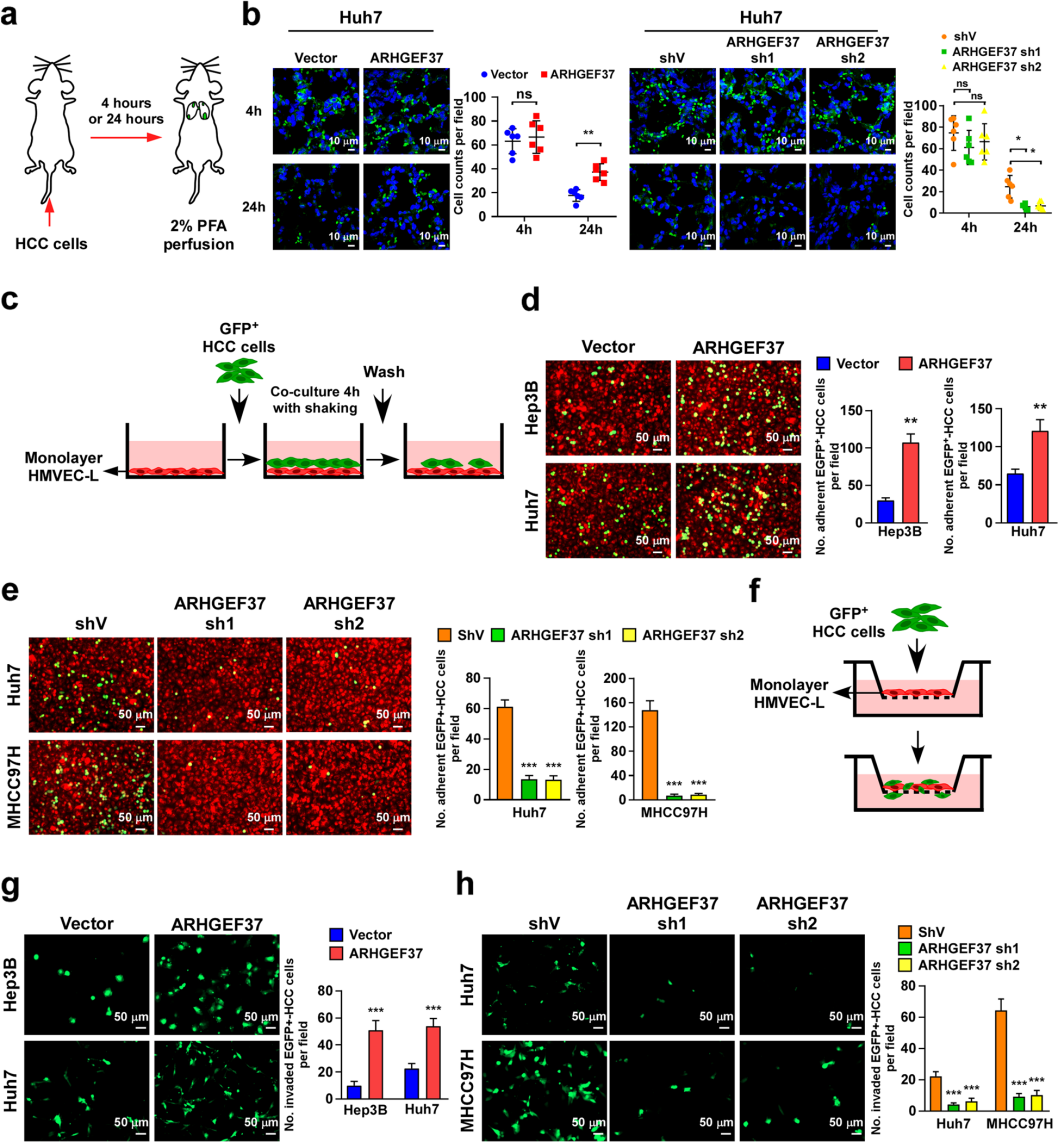
ARHGEF37 promotes endothelial adherence and trans-endothelial migration of tumor cells. **a** Schematic diagram of the extravasation assays. **b** The control, or ARHGEF37*-*overexpressing, or ARHGEF37-silenced GFP^+^-Huh7 cells were injected through tail veins. Lungs were assayed at 4 and 24 hours after injection (*n* = 6). Representative lung section images at 4 and 24 hours after injection are shown. The GFP^+^-HCC cells were detected and quantified. Scale bars: 10 μm. **c** Schematic diagram of the endothelial adhesion assays. **d** Representative immunofluorescence images and quantitation of the vector and HCC cells stably overexpressing ARHGEF37 in endothelial adhesion assays. Scale bars: 50 μm. **e** Representative immunofluorescence images and quantitation of ShV- and ARHGEF37-silenced stable HCC cells in endothelial adhesion assays. Scale bars: 50 μm. **f** Schematic diagram of the trans-endothelial migration assays. **g-h** Representative immunofluorescence images and quantitation of vector-, ARHGEF37 overexpressing- (**g**), and shV-, ARHGEF37-silenced (**h**) stable HCC cells in trans-endothelial migration assays. Scale bars: 50 μm. Each value in panel **b** represents the mean ± SD of six mice/group, and each error bar in panel **d**, **e**, **g**, and **h** represents the mean ± SD of three independent experiments. * *P* < 0.05; ** *P* < 0.01; *** *P* < 0.001; ns represents not significant

Lung metastasis involves adherence in the lung, extravasation, pre-metastatic niche formation, and the colonization and proliferation of tumor cells in the metastatic cascade [9]. To clarify which steps of metastasis were regulated by ARHGEF37, we performed lung dissection at 4 h or 24 h after the injection of GFP^+^-HCC cells through the tail vein in nude mice (Fig. 3a). As shown in Fig. 3b, equal numbers of HCC cells were detected in the lung sections at 4 h. However, compared with the vector-control cells, we observed a rapid loss of the ARHGEF37-silenced tumors cells, but not the ARHGEF37-overexpressing cells, in lung tissues at 24 h. This finding suggest that ARHGEF37 enhances adherence to the lung alveoli and the extravasation of tumor cells in the lung metastasis cascade.

To further verify the hypothesis that ARHGEF37 promotes HCC cell adherence to the lung alveoli, monolayer dynamic adhesion assay by seeding GFP^+^-HCC cells on a monolayer lung microvascular endothelial cell (HMVEC-L) followed by co-culture for 4 h with shaking (Fig. 3c) was performed. In line with above hypothesis, ARHGEF37 overexpression significantly facilitated the adhesion of cancer cells to HMVEC-L cells, whereas silencing ARHGEF37 impaired their adherence (Fig. 3d and e). Given the important role of endothelial adhesion in trans-endothelial migration [36], we further investigated the role of ARHGEF37 in the transmigration of HCC cells through endothelial cells (ECs) (Fig. 3f). As shown in Fig. 3g and h, the trans-endothelial migratory speed of HCC cells increased significantly when ARHGEF37 levels were upregulated, and decreased when ARHGEF37 levels were downregulated. Taken together, these results demonstrate that ARHGEF37 facilitates the adhesion to endothelial monolayers and the trans-endothelial migration of HCC cancer cells both in vivo and in vitro.

### ARHGEF37 directly interacts with and activates Cdc42

**Fig. 4:**
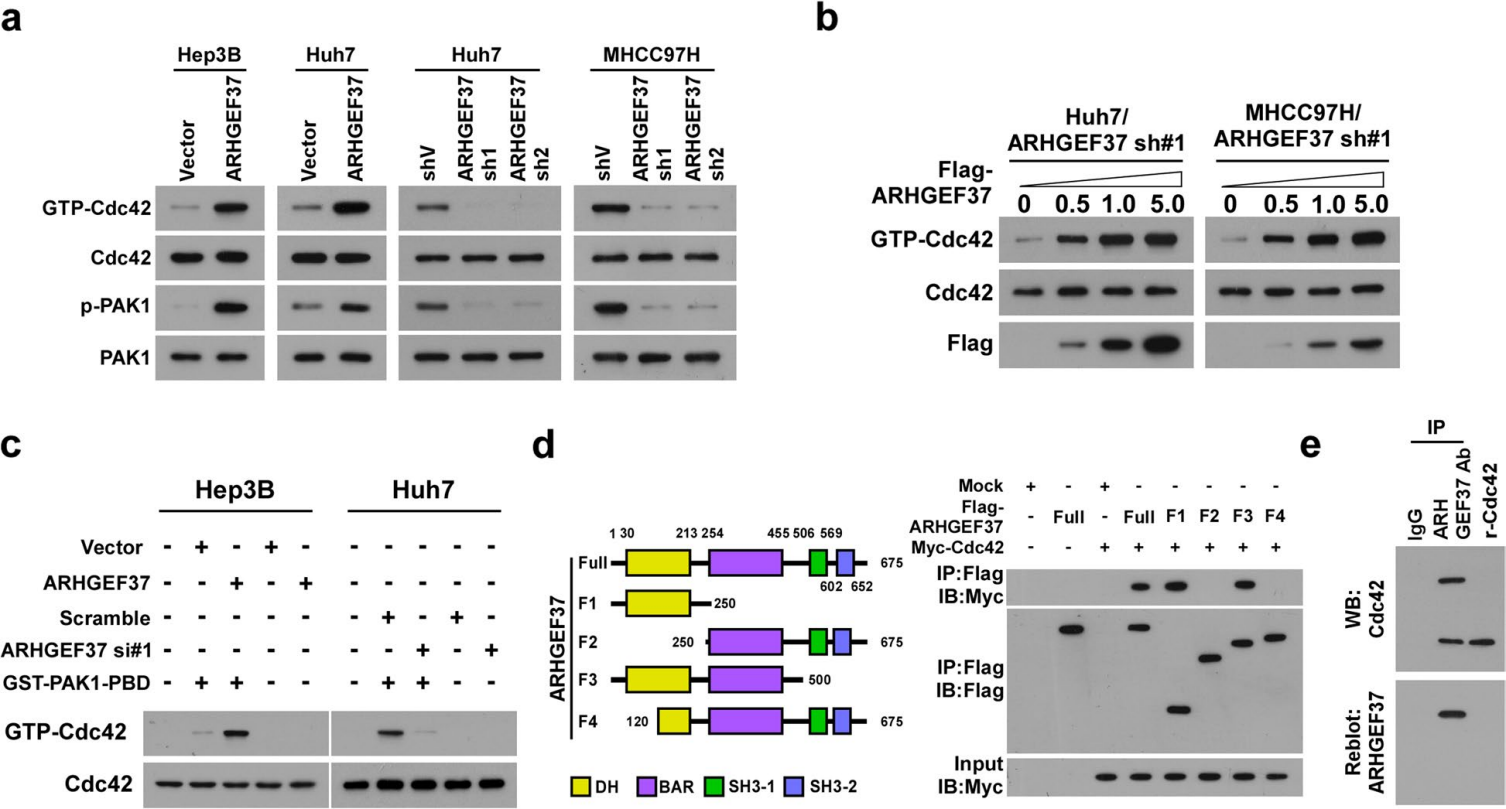
ARHGEF37 directly interacts with and activates Cdc42. **a** Immunoblotting analysis of GTP-Cdc42, Cdc42, p-PAK1, and PAK1 levels in the indicated cells. **b** Immunoblotting analysis of GTP-Cdc42, Cdc42, and Flag expression in the indicated cells. **c** Cdc42-GTP pull-down and western blotting analyses of the levels of activated Cdc42 and total Cdc42 in the indicated cells. **d** Schematic illustration of the wild-type and truncated ARHGEF37 protein (left); co-IP assays were performed using anti-Flag antibody in the indicated cells (right). **e** Far-western blotting analysis was performed using anti-ARHGEF37 antibody or anti-IgG antibody -immunoprecipitated proteins and detected using anti-Cdc42 antibody and then reblotted with anti-ARHGEF37 antibody. Recombinant Cdc42 protein served as the control. Data of panel **a**, **b**, **c**, **d**, and **e** are derived from three independent experiments

ARHGEF37 belongs to the guanine nucleotide exchange factor (GEF) family, which serve as small guanosine triphosphate phosphohydrolase (GTPase) activators via promoting the dissociation of GDP from GTPase and thereby facilitating GTP binding [15]. To determine the effect of ARHGEF37 on the regulation of GTPase activity, we first examined the levels of the GTP-bound forms of several GTPases, including Cdc42, Rac1, and RhoA, in ARHGEF37-transduced or silenced cells. Interestingly, we found that ARHGEF37 overexpression increased the level of the GTP-bound form of Cdc42 and the level of the phosphorylated downstream effector PAK1, but not the other two GTPases (Fig. 4a and Fig. S3a). Importantly, exogenous overexpression of ARHGEF37 markedly recovered the Cdc42 activity in ARHGEF37-silenced cells in a dose-dependent manner (Fig. 4b). Furthermore, an in vitro Cdc42 activation assay showed that overexpressing ARHGEF37 dramatically increased, whereas silencing ARHGEF37 decreased, the Cdc42 activity in HCC cells (Fig. 4c). Moreover, far-western blotting analysis indicated that ARHGEF37 directly bound to Cdc42 and Co-IP assays using serially truncated ARHGEF37 fragments showed that ARHGEF37 bound to Cdc42 through its Dbl homology (DH) domain (Fig. 4d and e). Therefore, our results demonstrate that ARHGEF37 directly interacts with and activates Cdc42.

### ARHGEF37 facilitates invadopodia formation in tumor cells and disrupts the interaction between ECs and pericytes

**Fig. 5:**
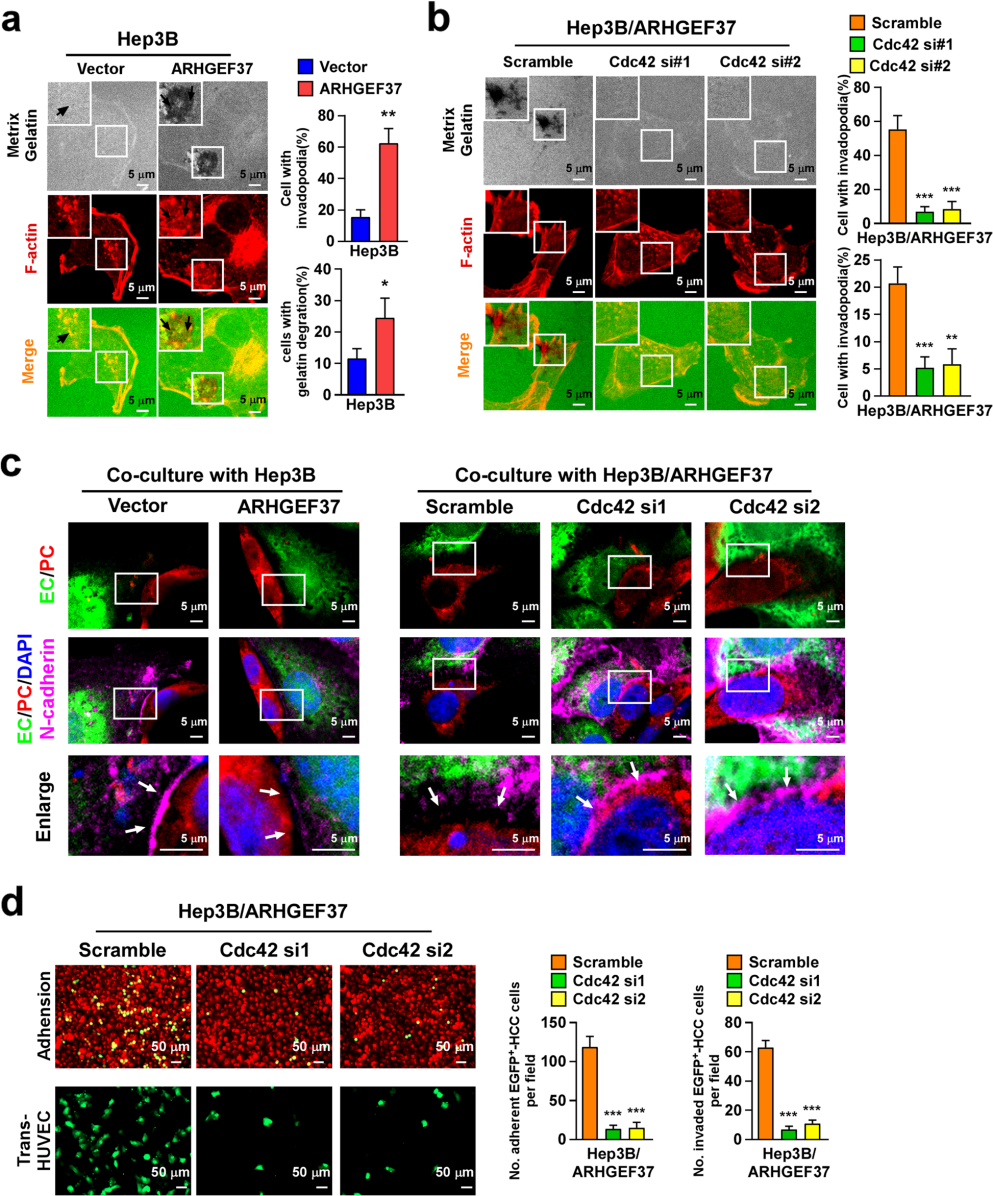
ARHGEF37 facilitates invadopodia formation in tumor cells and disrupts the interaction between endothelial cells and pericytes. **a** Hep3B cells (expressing vector or ARHGEF37) were plated on Fluorescein-conjugated gelatin (green) for 24 hours. F-actin was stained with phallodin (red) and nuclei with DAPI (blue). Areas of gelatin degradation appear as punctuate black areas beneath the cells. Scale bars: 5 μm. **b** Hep3B-ARHGEF37 cells expressing control or Cdc42 siRNA were seeded on Fluorescein-gelatin (green) for 24 hours and stained for F-actin (red) and nuclei (blue). Scale bars: 5 μm. **c** The pericyte-endothelial interaction in a 2D co-culture with the indicated tumor cells was analyzed by staining for N-cadherin. Scale bars: 5 μm. **d** Representative immunofluorescence images (upper) and quantitation (lower) of Hep3B-ARHGEF37 cells expressing control or Cdc42 siRNA in endothelial adhesion assays and in trans-endothelial migration assays. Scale bars: 50 μm. The data in panel **a**, **b** and **d** represents the mean ± SD of three independent experiments. *** *P* < 0.001

Cdc42 activation is closely related to the formation of invadopodia, which degrade the matrix [37, 38]. We then examined the effect of ARHGEF37 upregulation on the formation invadopodia and the degradation of the matrix using a fluorescent gelatin degradation assay. Interestingly, more foci of degraded matrix appeared upon ARHGEF37 overexpression, and these foci were enriched with the invadopodia marker F-actin (Fig. 5a). In contrast, the invadopodia formation induced by ARHGEF37 was substantially suppressed in cells with Cdc42 silencing (Fig. 5b). Therefore, these results suggest that ARHGEF37 plays a crucial role in the formation of invadopodia for cell invasion.

The results shown in Fig. 3a and b suggested that ARHGEF37 participated in the extravasation of tumor cells in the lung metastasis cascade. Pericytes are embedded within the endothelial basement membrane and directly interact with ECs at peg-socket-like structures containing tight-, gap-, and adherens junctions between the two cell types [39, 40]. To examine whether dysregulation of ARHGEF37 has affected on these intercellular junctions, we observed the pericyte–EC interaction in a 2D co-culture system with tumor cells. In the Hep3B-vector cell co-culture, pericytes and ECs established robust and continuous contacts, as shown by N-cadherin and Cx43 staining (Fig. 5c and Fig. S4a). In contrast, these contacts were disrupted, which displayed numerous intercellular gaps when co-cultured with ARHGEF37-transduced HCC cells. The defective pericyte–EC interaction was accompanied by a marked decrease in both N-cadherin and Cx43 accumulation at the cell–cell junctions (Fig. 5c and Fig. S4a). Moreover, silencing Cdc42 significantly abrogated the promotive effect of ARHGEF37 on adhesion and transmigration capabilities of HCC cells (Fig. 5d), suggested that the effect of ARHGEF37 on N-cadherin and Cx43-mediated pericyte–EC interaction was impaired under conditions of Cdc42 silencing.

We further examined the pericyte–EC interaction in vivo via triple immunostaining of EC marker CD31, pericyte marker PDGFRβ and the adherens junction protein N-cadherin, in the lung section of the mice bearing the metastatic foci. As shown in Fig. S4b, the ARHGEF37-overexpressing mice displayed substantial reduced association of PDGFRβ^+^ pericytes with capillaries, accompanying with attenuated N-cadherin signal, compared with the control mice. Meanwhile, the pericytes completely enveloped around the vasculature in ARHGEF37-silenced mice, whereas the control mice exhibited patchy coverage the pericytes that were less connected with the endothelial cells (Fig. S4c). Taken together, these results provide further evidence that ARHGEF37 disrupts the interaction between ECs and pericytes via Cdc42.

### Inhibition of Cdc42 activity blocks the effects of ARHGEF37 on tumor cell invasion

**Fig. 6:**
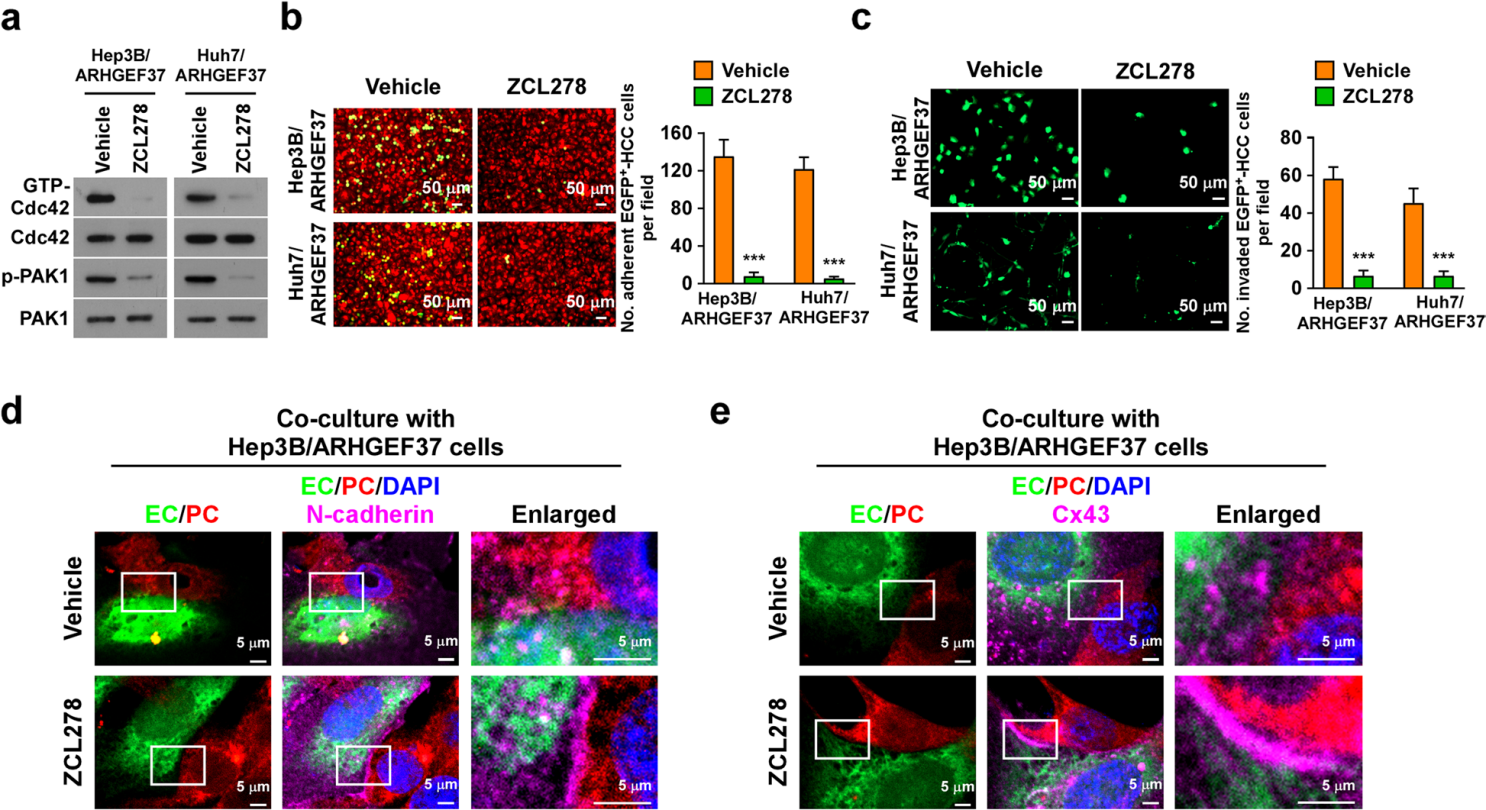
Inhibition of Cdc42 activity blocks the effects of ARHGEF37 on invasiveness. **a** Immunoblotting analysis of GTP-Cdc42, Cdc42, p-PAK1, and PAK1 levels in the indicated cells treated with vehicle or a Cdc42 specific inhibitor, ZCL278. **b** Representative immunofluorescence images and quantitation of ARHGEF37-transduced cells treated with vehicle or ZCL278 in endothelial adhesion assays. Scale bars: 50 μm. **c** Representative immunofluorescence images and quantitation of ARHGEF37-transduced cells treated with vehicle or ZCL278 in trans-endothelial migration assays. Scale bars: 50 μm. **d** The pericyte-endothelial interaction junctional protein N-cadherin was analyzed in a 2D co-culture with ARHGEF37-transduced tumor cells upon treatment with vehicle or ZCL278. Scale bars: 5 μm. **e** The pericyte-endothelial interaction junctional protein Cx43 was analyzed in a 2D co-culture with ARHGEF37-transduced cells tumor cells upon treating with vehicle or ZCL278. Scale bars: 5 μm. Data of panel **a** are derived from three independent experiments. Each error bar in panel **b** and **c** represents the mean ± SD of three independent experiments. * *P* < 0.05; ** *P* < 0.01; *** *P* < 0.001

Given that ARHGEF37 is associated with EC adherence and trans-endothelial migration via regulating Cdc42 activity, we speculated that a Cdc42 inhibitor would be potential therapeutic agent for ARHGEF37-mediated tumor cell invasion. ZCL278 is a selective Cdc42 inhibitor that inhibits Cdc42’s GTPase activity by competing with GTP [41]. As shown in Fig. 6a, ZCL278 significantly inhibited the effect of ARHGEF37 on activation of Cdc42 and the downstream effector PAK1 in ARHGEF37-overexpressing Hep3B and Huh7 cells. Furthermore, ZCL278 treatment interfered with ARHGEF37-transduced tumor cell adhesion to an EC monolayer compared that of with the vehicle group (Fig. 6b). Prominently, ZCL278 inhibited the trans-endothelial migration ability induced by ARHGEF37 overexpression (Fig. 6c), where the rescued pericyte–EC interaction was accompanied by a marked increase in both N-cadherin and Cx43 accumulation at the cell–cell junctions (Fig. 6d and e). Thus, pharmacological blocking of Cdc42 could inhibit the pro-invasion effect of ARHGEF37.

### Pharmacological blocking of Cdc42 inhibits pulmonary metastasis of HCC in vivo

**Fig. 7:**
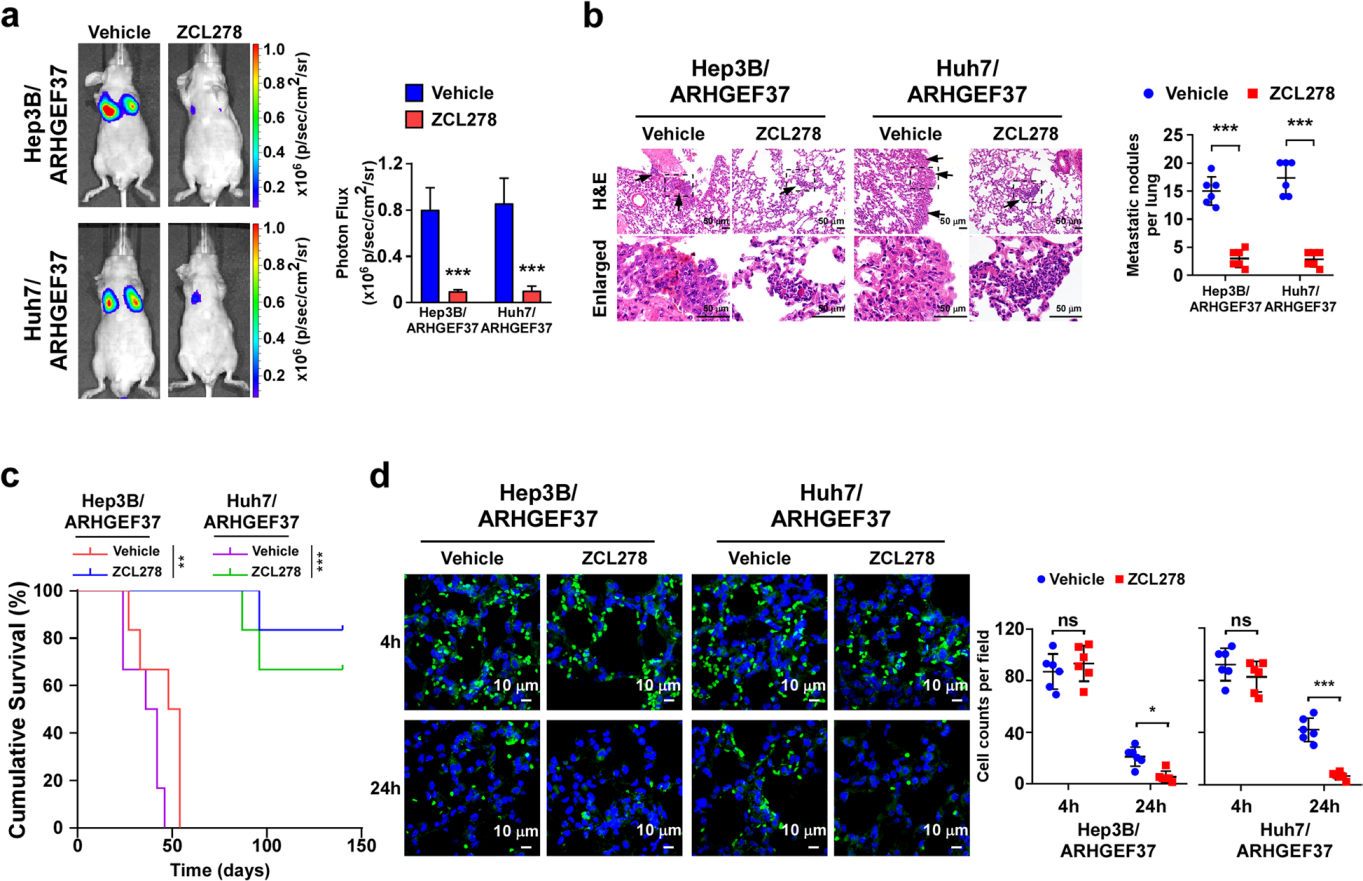
Pharmacological blocking of Cdc42 inhibits pulmonary metastasis of HCC in vivo. **a** Left: Representative bioluminescence images of pulmonary metastasis from vehicle- or ZCL278-treated mice (*n* = 6/group) tail vein intravenously injected with HCC cells stably overexpressing ARHGEF37. Right: The statistical results of the luminescence signals for lung metastasis. **b** Representative images and quantitation of metastatic nodules in the lungs. Scale bars: 50 μm. **c** Kaplan–Meier survival curve of mice from experiments in (**a**) (*n* = 6/group). **d** The control or ARHGEF37-overexpressing GFP^+^-HCC cells were treated with vehicle or ZCL278 before injection through tail veins. Lungs were assayed at 4 and 24 hours after injection (*n* = 6). Representative lung section images at 4 and 24 hours after injection are shown. The GFP^+^-HCC cells were detected and quantified. Scale bars: 10 μm. Each value in panel **a, b and d** represents the mean ± SD of six mice/group. * *P* < 0.05; ** *P* < 0.01; *** *P* < 0.001; ns represents not significant

Finally, we examined whether pharmacological inhibition of Cdc42 activity by ZCL278 could repress pulmonary metastasis of HCC. As shown in Fig. 7a, the ARHGEF37-transduced HCC cells-injected mice treated with ZCL278 exhibited weaker signals of lung metastasis and reduced numbers of lung metastatic nodules (Fig. 7a, b and Fig. S5a). Moreover, treatment of ZCL278 in the mice xenografted with HCC cells significantly prolonged their overall survival (Fig. 7c). Consistently, equal numbers of tumor cells were detected in the lung sections at 4 h, followed by a rapid loss in the ZCL278-treated group, but not in the vehicle group, in lung tissues at 24 h. This finding suggested that treatment with ZCL278 impaired tumor cell adherence in the lung alveoli and their extravasation. During the treatment course, ZCL278-treated mice did not exhibit obvious weight loss, diarrhea, anorexia, skin ulceration and general non-well-being, as well as alterations of the alanine aminotransferase (ALT) and aspartate aminotransferase (AST) levels, compared with the vehicle-treated mice, which indicated that the dosage of ZCL278 used in the current study had no significant side effects. Taken together, our results demonstrate that the ARHGEF37-Cdc42 axis plays an important role in invasiveness of HCC cells and represents a potential therapeutic target for pulmonary metastasis in HCC.

## Discussion

Metastasis is the most frequent distant invasive progression and is one of the main causes of cancer-related death in HCC [42]. Some of the available anti-tumor therapies aimed at the inhibition of primary tumor growth also affect the growth of metastasis [3, 42]. If HCC metastasis is limited to the liver, curative surgical therapies, such as surgical resection or orthotopic liver transplantation, might be offered [43]. However, if extrahepatic spread occurs, the patient will be left with only palliation because of the lack of effective interventions against HCC extrahepatic metastasis. Thus, investigations of the molecular mechanisms underlying HCC metastasis are urgently needed to develop potential targeted therapeutic strategies. Importantly, the most common site of metastasis is the lung, accounting for 34–58% of cases [7]. To address the mechanism underlying pulmonary metastasis, we found that ARHGEF37-mediated Cdc42 activation plays a vital role in lung metastasis via modulation of the adhesion and extravasation of tumor cells. Importantly, ZCL278, the specific inhibitor of Cdc42, decreased the level of activated Cdc42 and effectively inhibited co-localization and further progression of HCC lung metastasis. Therefore, these findings reveal a molecular circuit for HCC lung metastasis and may provide new therapeutic targets to treat metastatic disease.

Our results showed that more GFP^+^-HCC cells were observed in the lung alveoli in the lung dissection at 24 h after intravenous injection of the ARHGEF37-transduced cells compared with that in the vector group, which was consistent with the finding that the metastatic potential of tumor cells ultimately depends on their ability to rapidly extravasate into the surrounding tissue [14, 44, 45]. Extravasation involves adhesion and trans-endothelial migration into the endothelium [9, 10], and appears to mainly entail the disruption of inter-endothelial cell-cell junctions [14]. During extravasation, Rho GTPase signaling networks not only regulate the adhesion of cancer cells [11], but also regulate cancer cell transmigration and are involved in the formation of invadopodia to disrupt the endothelial barrier function [14], ultimately allowing cancer cells to extravasate into the underlying tissue and potentially form metastases. In the present study, we demonstrated that ARHGEF37-mediated Cdc42 activation promoted tumor cell adhesion and further extravasation across the monolayer of the endothelium by enhancing the formation of invadopodia. Inhibiting Cdc42 expression by RNAi markedly decreased the number of invadopodia and the extent of matrix degradation, and repressed the adherence and trans-endothelial migration ability of ARHGEF37-transduced HCC cells. Consistently, treatment with the pharmacological inhibitor ZCL278 significantly inhibited the adherence, extravasation, and further lung metastasis mediated by ARHGEF37 overexpression.

To reach the distant surrounding stroma, tumor cells need to spread across the abluminal surface of blood vessels, which is encapsulated by pericytes that form direct contacts with ECs. This spreading might require the disruption of the pericyte-EC interaction, which is highly enriched with N-cadherin-dependent adherens junctions and Cx43-dependent gap junctions [46]. Consistent with this hypothesis, we found that co-culture with ARHGEF37-overexpressing HCC cells impaired the N-cadherin and Cx43-mediated pericyte-EC interaction. Alternatively, blocking the activation of Cdc42 significantly restored the pericyte-EC interaction. Considering the ability of invadopodia to degrade the extracellular matrix, the destruction the pericyte-EC interaction might also be regulated by the formation of invadopodia induced by ARHGEF37/Cdc42-GTP. Thus, our results unveil a plausible mechanism of ARHGEF37 in lung metastasis, placing ARHGEF37 at the focal point for the prediction and prevention of HCC lung metastasis.

During the process of extravasation, cancer cells need to continuously change their shape to adhere and transmigrate through the endothelium. These processes require remodeling of the actin cytoskeleton, which is centrally regulated by Rho GTPase signaling networks [11, 36]. The activity of the Rho GTPases is determined by their GTP/GDP-bound forms in the cell, in which the ratio of the two forms is regulated by the opposing effects of GEFs and GTPase-activating proteins [36]. Accumulating evidence has demonstrated that the majority of GEFs specific for Rho GTPases can promote cancer cell invasion and metastasis by enhancing the exchange of GTP onto Rho proteins [47, 48]. In the present study, we found the upregulation of ARHGEF37, a potential GEF, increased the level of the GTP bound-form of Cdc42 via direct binding with Cdc42 through its DH domain. It is assumed that Cdc42 activation induces the formation of filopodia and invadopodia to promote cancer progression by enhancing the acquisition of migratory and invasive properties in tumor cells. Therefore, we hypothesized that ARHGEF37 overexpression-mediated Cdc42 activation might play an important role in HCC lung metastasis. To confirm our hypothesis, we performed both in vitro and in vivo assays, the results of which demonstrated that overexpression of ARHGEF37 led to an increased adhesion and trans-endothelial migration capability of tumor cells and invadopodia formation. Moreover, these invasion- and metastasis-associated phenotypes could be effectively abolished by silencing Cdc42 expression in HCC cells overexpressing ARHGEF37. These finding strongly suggest that the metastatic function of ARHGEF37 occurs via activation of Cdc42.

## Conclusions

In summary, our study demonstrate that the ARHGEF37-Cdc42 axis is clinically and functionally associated with pulmonary metastasis of HCC. These findings not only illustrate the invasive and metastatic mechanism of ARHGEF37 in pulmonary metastasis, but also provide new therapeutic targets for the prevention and treatment of HCC pulmonary metastasis.

## Supplementary Information

**Additional file 1: Table S1**. Clinicopathological characteristics of studied patients and expression of ARHGEF37 in HCC. **Table S2**. Correlation between the clinicopathological features and expression of ARHGEF37. **Table S3.** Univariate and multivariate analysis of factors associated with Overall Survival in 365 HCC patients. **Table S4.** Univariate and multivariate analysis of factors associated with Metastasis-free Survival in 365 HCC patients. **Table S5.** Sequence of primers used for subcloning and plasmid construction. **Table S6.** Sequence of primers used for qPCR. **Table S7.** Sequence of Oligonucleotides for siRNAs. **Figure S1.** *ARHGEF37* is upregulated in HCC. (**a**) Analysis of TCGA datasets showing that *ARHGEF37* was upregulated in HCC tumor tissues compared with that in normal liver tissues (*P* < 0.001). **Figure S2.** ARHGEF37 overexpression promotes pulmonary metastasis in HCC. **Figure S3.** ARHGEF37 did not induce the overexpression of GTP-bound form of Rac1 and RhoA. **Figure S4.** ARHGEF37 facilitates invadopodia formation in tumor cells and disrupts the interaction between endothelial cells and pericytes. **Figure S5.** In vivo mouse experiments where tumors metastasis was tracked by bioluminescence imaging.

## Data Availability

The data supporting the conclusions of this article are provided in this article and the additional files. In addition, all data from this study can be obtained from the corresponding author upon reasonable request.

## Abbreviations

ARHGEF37: Rho guanine nucleotide exchange factor 37
Cdc42: Cell division cycle 42
HCC: Hepatocellular carcinoma
Rho-GEFs: Rho family guanine nucleotide exchange factors
HEK: Human embryonic kidney cells
DMEM: Dulbecco’s modified Eagle’s medium
STR: Short tandem repeat
shRNAs: Short hairpin RNAs
siRNAs: Small interfering RNAs
PBS: Phosphate-buffered saline
EGFP: Enhanced green fluorescent protein
qPCR: Quantitative real time PCR
GAPDH: Glyceraldehyde-3-phosphate dehydrogenase
Cx43: Connexin-43
ECs: Epithelial cells
DAPI: 4,6-diamidino-2-phenylindole
SD: Standard deviation
HMVEC-L: Lung microvascular endothelial cells
GEF: Guanine nucleotide exchange factor
GTPase: Guanosine triphosphate phosphohydrolase
DH: Dbl homology
