# Supplementary material for: ARHGEF37 overexpression promotes extravasation and metastasis of hepatocellular carcinoma via directly activating Cdc42
Source: J Exp Clin Cancer Res. 2022 Jul 22;41:230. doi: 10.1186/s13046-022-02441-y (PMC9308268; doi:10.1186/s13046-022-02441-y)
Supplement: Supplementary file 1 — Additional file 1: Table S1. Clinicopathological characteristics of studied patients and expression of ARHGEF37 in HCC. Table S2. Correlation between the clinicopathological features and expression of ARHGEF37. Table S3. Univariate and multivariate analysis of factors associated with Overall Survival in 365 HCC patients. Table S4. Univariate and multivariate analysis of factors associated with Metastasis-free Survival in 365 HCC patients. Table S5. Sequence of primers used for subcloning and plasmid construction. Table S6. Sequence of primers used for qPCR. Table S7. Sequence of Oligonucleotides for siRNAs. Figure S1. ARHGEF37 is upregulated in HCC. (a) Analysis of TCGA datasets showing that ARHGEF37 was upregulated in HCC tumor tissues compared with that in normal liver tissues (P < 0.001). Figure S2. ARHGEF37 overexpression promotes pulmonary metastasis in HCC. Figure S3. ARHGEF37 did not induce the overexpression of GTP-bound form of Rac1 and RhoA. Figure S4. ARHGEF37 facilitates invadopodia formation in tumor cells and disrupts the interaction between endothelial cells and pericytes. Figure S5. In vivo mouse experiments where tumors metastasis was tracked by bioluminescence imaging. [file 13046_2022_2441_MOESM1_ESM.docx]

Additional files

**Supplementary Tables**

**Table S1**. Clinicopathological characteristics of studied patients and expression of ARHGEF37 in HCC

| **Factor** | **No.** | **(%)** |
| --- | --- | --- |
| **Gender** |  |  |
| Male | 324 | 88.8 |
| Female | 41 | 11.2 |
| **Age (years)** |  |  |
| ≤60 | 192 | 52.6 |
| >60 | 173 | 47.4 |
| **Clinical stage** |  |  |
| I-II | 176 | 48.2 |
| III-IV | 189 | 51.8 |
| **HBsAg** |  |  |
| Positive | 325 | 89.0 |
| Negative | 40 | 11.0 |
| **Tumor size(cm) differentiation** |  |  |
| ≤5cm | 190 | 52.1 |
| >5cm | 175 | 47.9 |
| **Extrahepatic metastasis** |  |  |
| No | 70 | 60.9 |
| Yes | 45 | 39.1 |
| **Vital status** |  |  |
| Alive | 168 | 46.0 |
| Dead | 197 | 54.0 |
| **Expression of ARHGEF37** |  |  |
| Low expression | 189 | 51.8 |
| High expression | 176 | 48.2 |

AFP, α-fetoprotein; HBsAg, hepatitis B antigen.

**Table S2**. Correlation between the clinicopathological features and expression of ARHGEF37

|  |  | **ARHGEF37 expression** | |  |
| --- | --- | --- | --- | --- |
| **Parameters** | **Number of cases** | **Low (n=189)** | **High (n=176)** | ***P* values** |
| **Gender** |  |  |  |  |
| Male | 324 | 168 | 156 | 1.000 |
| Female | 41 | 21 | 20 |  |
| **Age (years)** |  |  |  |  |
| ≤60 | 192 | 97 | 95 | 0.675 |
| >60 | 173 | 92 | 81 |  |
| **Clinical stage** |  |  |  |  |
| I-II | 176 | 87 | 89 | 0.403 |
| III-IV | 189 | 102 | 87 |  |
| **HBsAg** |  |  |  |  |
| Positive | 325 | 170 | 155 | 0.617 |
| Negative | 40 | 19 | 21 |  |
| **Tumor size(cm)** |  |  |  |  |
| ≤5 | 190 | 117 | 73 | <0.001 |
| >5 | 175 | 72 | 103 |  |
| **Extrahepatic metastasis** |  |  |  |  |
| No | 70 | 34 | 36 | 0.021 |
| Yes | 45 | 12 | 33 |  |
| **Survival status** |  |  |  |  |
| Alive | 168 | 106 | 62 | <0.001 |
| Dead | 197 | 83 | 114 |  |

**Table S3.** Univariate and multivariate analysis of factors associated with Overall Survival in 365 HCC patients.

| Characteristics | Univariate analysis | | Multivariate analysis | |
| --- | --- | --- | --- | --- |
|  | HR (95% CI) | *P* values | HR (95% CI) | *P* values |
| **Age (years)** | 0.963 (0.728-1.275) | 0.792 | 1.109 (0.834-1.476) | 0.439 |
| **Gender** | 0.987 (0.628-1.552) | 0.955 | 0.973 (0.617-1.534) | 0.865 |
| **Clinical stages** | 1.699  (1.276 -2.262) | <0.001 | 1.712 (1.281-2.287) | <0.001 |
| **HBsAg** | 0.772 (0.510-1.167) | 0.219 | 0.806 (0.531-1.224) | 0.292 |
| **Tumor sizes** | 1.146 (0.867-1.516) | 0.338 | 1.018 (0.765-1.355) | 0.861 |
| **ARHGEF37 expression** | 3.624 (2.634-4.986) | < 0.001 | 3.012 (2.229-4.070) | <0.001 |

HR, hazard ratio; CI, confidence interval.

**Table S4.** Univariate and multivariate analysis of factors associated with Metastasis-free Survival in 365 HCC patients.

| Characteristics | Univariate analysis | | Multivariate analysis | |
| --- | --- | --- | --- | --- |
|  | HR (95% CI) | *P* values | HR (95% CI) | *P* values |
| **Age (years)** | 1.120 (0.622-2.017) | 0.705 | 1.150 (0.630-2.098) | 0.644 |
| **Gender** | 0.633 (0.282-1.423) | 0.269 | 0.721 (0.545-1.745) | 0.450 |
| **Clinical stages** | 0.855  (0.468-1.563) | 0.611 | 0.765 (0.408-1.434) | 0.404 |
| **HBsAg** | 1.857 (0.575-5.996) | 0.300 | 2.247 (0.679-7.438) | 0.184 |
| **Tumor sizes** | 1.091 (0.605-1.966) | 0.773 | 0.882 (0.447-1.552) | 0.569 |
| **ARHGEF37 expression** | 3.639 (1.793-7.387) | < 0.001 | 4.047 (1.946-8.417) | < 0.001 |

HR, hazard ratio; CI, confidence interval.

**Table S5. Sequence of primers used for subcloning and plasmid construction**

| **Subcloning and plasmid construction:** | **Primers** |
| --- | --- |
| pSin-EF2-puro-  ARHGEF37-Flag-forward | 5’-GGATCCCCGGACGAATTCATGGATTACAAGGATGACGACGATAAGGCCAAGCATGGAGCC -3’ |
| pSin-EF2-puro-  ARHGEF37-Flag-reverse | 5’-TCATATGTTCGAAGAATTCTCACTAAGAGGGCAGACTCCAGCCCCACAGAACTGGGCTCCG -3’ |
| pSuper Retro-  ARHGEF37-RNAi#1-forward | 5’-GATCCCCGCGTGGGTCACACGGGATAATTTCAAGAGAATTATCCCGTGTGACCCACGCTTTTTA-3’ |
| pSuper Retro-  ARHGEF37-RNAi#1-reverse | 5’-AGCTTAAAAAGCGTGGGTCACACGGGATAATTCTCTTGAAATTATCCCGTGTGACCCACGCGGG-3’ |
| pSuper Retro-  ARHGEF37-RNAi#2-forward | 5’-GATCCCCGGAACAAGTGCAGCTAGTTTTCAAGAGAAACTAGCTGCACTTGTTCCTTTTTA -3’ |
| pSuper Retro-  ARHGEF37-RNAi#2-reverse | 5’-AGCTTAAAAAGGAACAAGTGCAGCTAGTTTCTCTTGAAAACTAGCTGCACTTGTTCCGGG -3’ |

**Table S6. Sequence of primers used for** **qPCR**

| **Genes** | **Primers** |
| --- | --- |
| ARHGEF37-forward | 5’- AGGAACAAGTGCAGCTAGTTG-3’ |
| ARHGEF37-reverse | 5’- GCTGGCACAGTAGACCTTATAGA-3’ |
| Cdc42-forward | 5’- CCATCGGAATATGTACCGACTG-3’ |
| Cdc42-reverse | 5’- CTCAGCGGTCGTAATCTGTCA-3’ |

**Table S7. Sequence of Oligonucleotides for siRNAs**

| **Genes** | **siRNAs** |
| --- | --- |
| ARHGEF37#1 siRNA | GCGTGGGTCACACGGGATAAT |
| ARHGEF37#2 siRNA | GGAACAAGTGCAGCTAGTT |
| Cdc42#1 siRNA | AAGTGGGTGCCTGAGATAACT |
| Cdc42#2 siRNA | AAAGACTCCTTTCTTGCTTGT |

**Supplementary Figures**

**
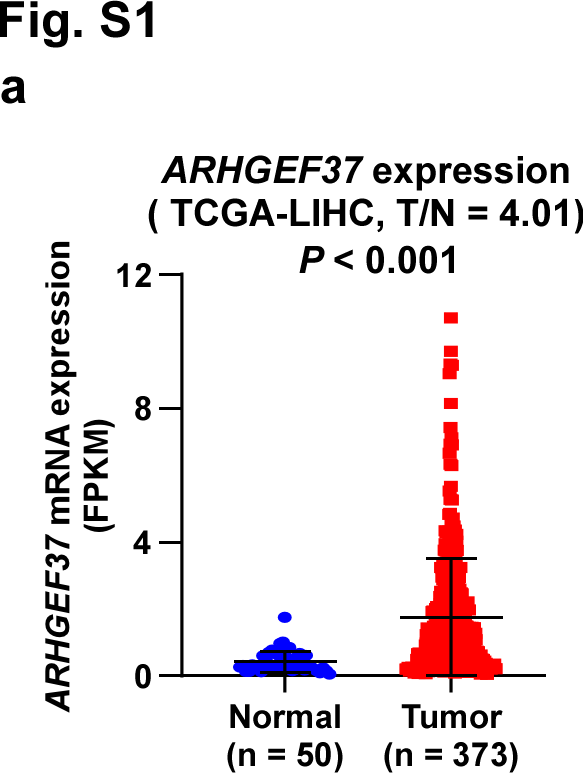
**

**Fig. S1. *ARHGEF37* is upregulated in HCC.** (**a**) Analysis of TCGA datasets showing that *ARHGEF37* was upregulated in HCC tumor tissues compared with that in normal liver tissues (*P* < 0.001).

**
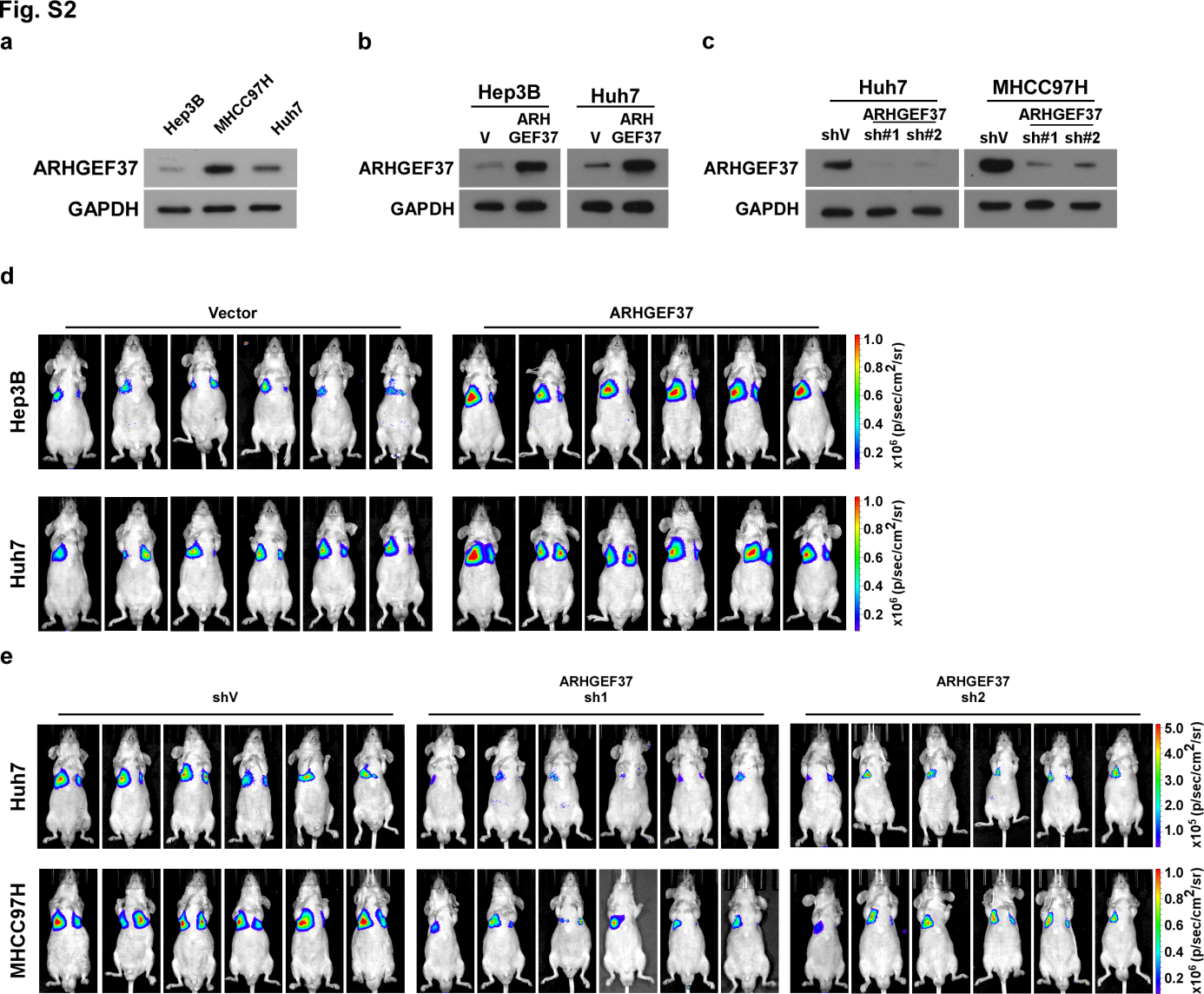
**

**Fig. S2. ARHGEF37 overexpression promotes pulmonary metastasis in HCC.** (**a**) Immunoblotting analysis of ARHGEF37 levels in Hep3B, Huh7 and MHCC97H cells. GAPDH served as the loading control. (**b**) Immunoblotting analysis of ARHGEF37 levels in vector- and ARHGEF37- transduced Hep3B and Huh7 cells. GAPDH served as the loading control. (**c**) Immunoblotting analysis of ARHGEF37 levels in shv- and ARHGEF37-shRNA transduced Huh7 and MHCC97H cells. GAPDH served as the loading control. (**d**) Representative bioluminescence images of pulmonary metastasis in mice after tail vein intravenous injection with vector and HCC cells stably overexpressing ARHGEF37. The color scale bar depicts the photon flux emitted from the mice and pulmonary metastasis (n = 6). (**e**) Representative bioluminescence images of pulmonary metastasis in mice after tail vein intravenous injection with shV- and stable ARHGEF37-silenced HCC cells. The color scale bar depicts the photon flux emitted from the mice and pulmonary metastasis (n = 6). Data of panel **a, b, and c** are derived from three independent experiments.

**
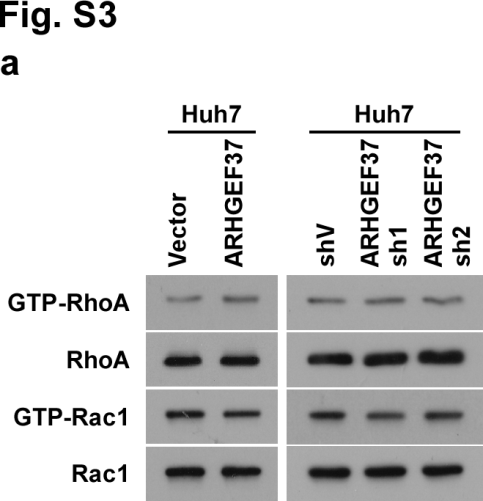
**

**Fig. S3. ARHGEF37 did not induce the overexpression of GTP-bound form of Rac1 and RhoA.** (**a**) Immunoblotting analysis of GTP-RhoA, RhoA, GTP-Rac1 and Rac1 levels in the indicated cells. Data of panel **a** is derived from three independent experiments.

**
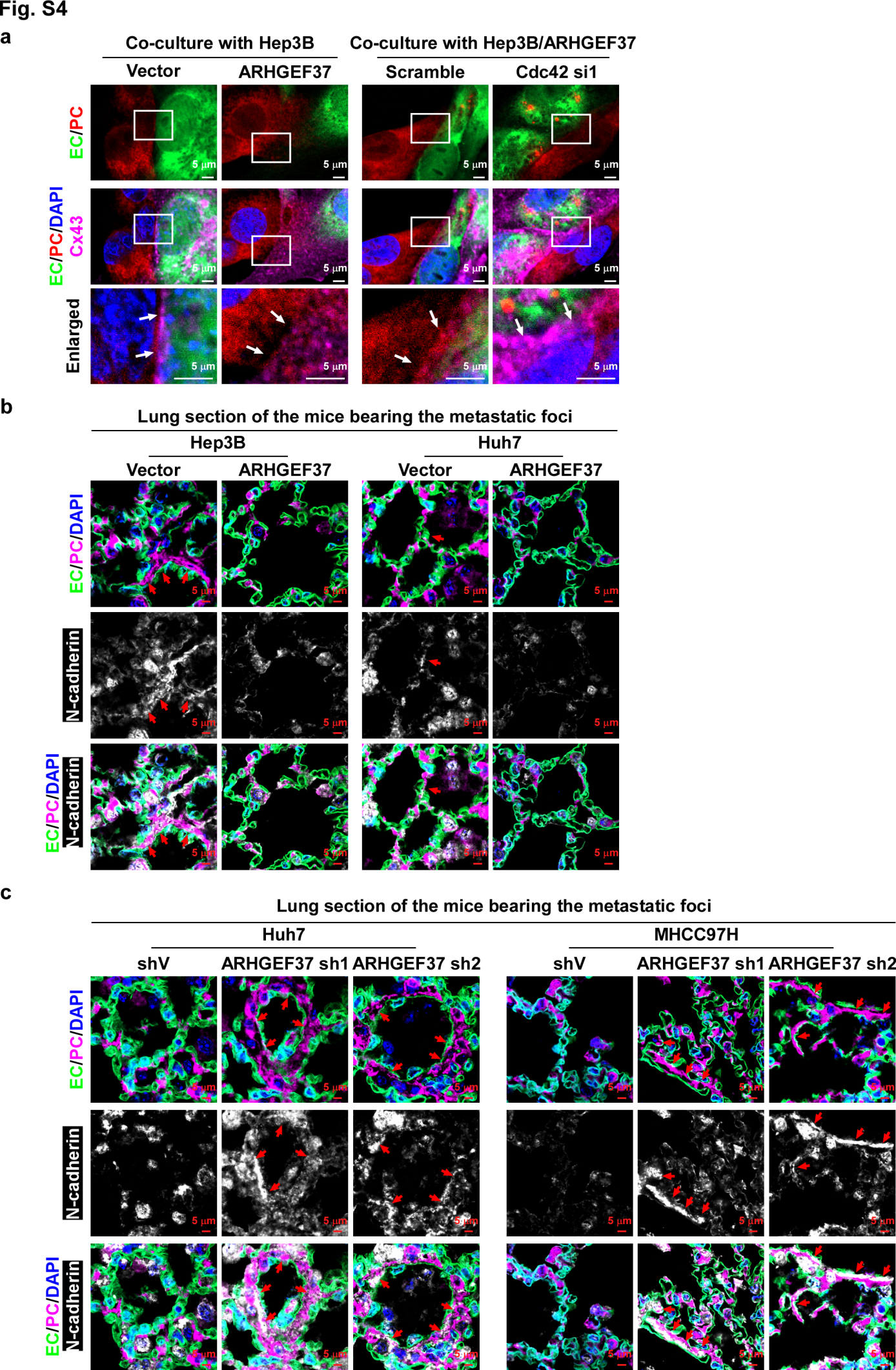
**

**Fig. S4. ARHGEF37 facilitates invadopodia formation in tumor cells and disrupts the interaction between endothelial cells and pericytes.** (**a**) The pericyte-endothelial interaction in a 2D co-culture with the indicated tumor cells was analyzed by staining for Cx43. Scale bars: 5 µm. (**b-c**) The triple immunostaining of EC marker CD31, pericyte marker PDGFRβ and the adherens junction protein N-cadherin in the lung section of the mice bearing the metastatic foci. Scale bars: 5 µm. Data of panel **a, b, c** are derived from three independent experiments.


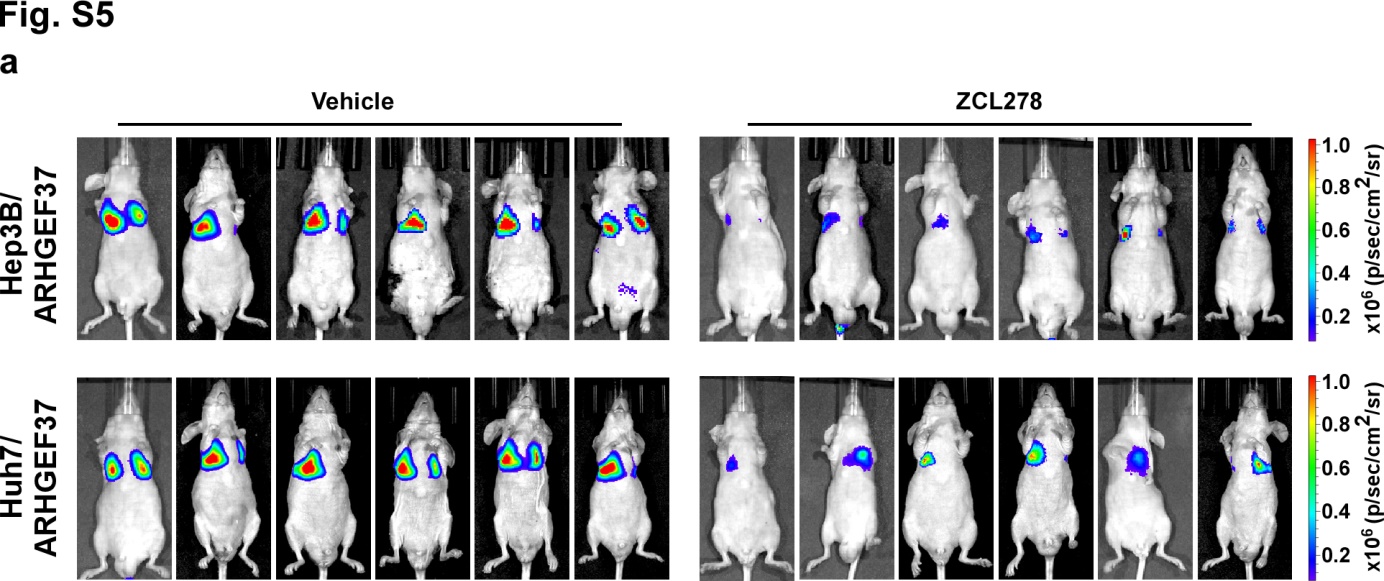


**Fig. S5. *In vivo* mouse experiments where tumors metastasis was tracked by bioluminescence imaging.** (**a**) Representative bioluminescence images of pulmonary metastasis from vehicle- or ZCL278-treated mice (n = 6/group) tail vein intravenously injected with HCC cells stably overexpressing ARHGEF37.
